# FM-UWB: Towards a Robust, Low-Power Radio for Body Area Networks

**DOI:** 10.3390/s17051043

**Published:** 2017-05-06

**Authors:** Vladimir Kopta, John Farserotu, Christian Enz

**Affiliations:** 1 Integrated Circuits Lab (ICLAB), Swiss Federal Institute of Technology (EPFL), Microcity, Rue de la Maladiére 71,2000 Neuchâtel, Switzerland; christian.enz@epfl.ch; 2 Swiss Center for Electronics and Microtechnology (CSEM), Rue Jaquet-Droz 1, 2000 Neuchâtel, Switzerland; john.farserotu@csem.ch

**Keywords:** UWB, FM, low-power, Wireless Body Area Networks (WBAN), Frequency Division Multiple Access (FDMA), multi-user, transceiver

## Abstract

The Frequency Modulated Ultra-Wideband (FM-UWB) is known as a low-power, low-complexity modulation scheme targeting low to moderate data rates in applications such as wireless body area networks. In this paper, a thorough review of all FM-UWB receivers and transmitters reported in literature is presented. The emphasis is on trends in power reduction that exhibit an improvement by a factor 20 over the past eight years, showing the high potential of FM-UWB. The main architectural and circuit techniques that have led to this improvement are highlighted. Seldom explored potential of using higher data rates and more complex modulations is demonstrated as a way to increase energy efficiency of FM-UWB. Multi-user communication over a single Radio Frequency (RF) channel is explored in more depth and multi-channel transmission is proposed as an extension of standard FM-UWB. The two techniques provide means of decreasing network latency, improving performance, and allow the FM-UWB to accommodate the increasing number of sensor nodes in the emerging applications such as High-Density Wireless Sensor Networks.

## 1. Introduction

**Figure 1:**
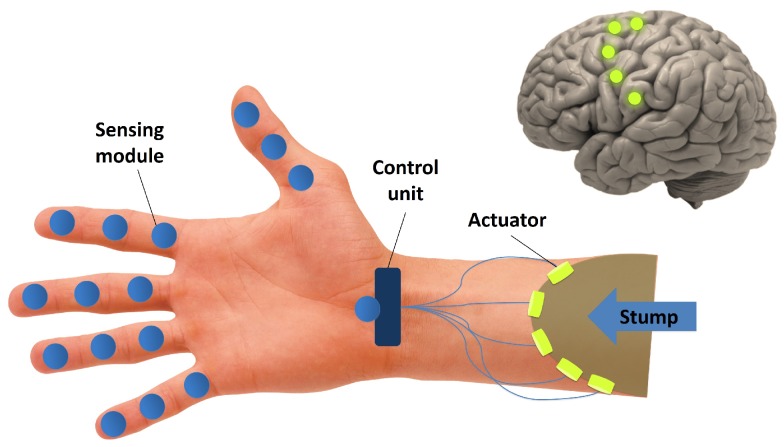
Illustration of the *WiseSkin* concept, wireless sensor nodes embedded in an artificial skin provide a sense of touch to the patient.

The recent advances in circuit design, combined with technology scaling, enabled highly miniaturized, ultra-low power radios that have been the key driving force behind the development of Wireless Body Area Networks (WBANs). These highly localized wireless networks find use in various medical and consumer applications [1]. In order to allow for the unobtrusive use, the size and the battery life of WBAN nodes are critical issues. The need for frequent replacement is undesirable and in the case of implanted nodes unacceptable, which sets the limit to the power consumption of the devices used in sensor nodes, in particular the radios, as they are often identified as the dominant source of power consumption. With the increasing popularity of WBAN, the number of applications continues to grow, as well as the number of connected devices. Furthermore, some applications require that a large number of devices coexist in a small area. One example is the *WiseSkin* concept (Figure 1), where sensor nodes embedded in a kind of artificial skin are placed on top of a prosthetic arm. The nodes communicate wirelessly between each other and transmit the information from sensors to the central processing unit that drives the actuators and provides a sense of touch to the patient [2]. The *WiseSkin* application is one example of a High-Density Wireless Sensor Network (HD-WSN). To deal with a large number of nodes operating in a small area, new protocols are needed, and a communication system that could support Frequency Division Multiple Access (FDMA) and multi-user communication is highly desirable. The short distance between adjacent nodes can be exploited to save power as it loosens the sensitivity constraints.

The designers are now faced with more and more challenges in an attempt to implement miniature radios that will not only consume very little power but also be capable of providing robust communication while dealing with the increasing number of interferers in the crowded spectrum of the future. The two Ultra-Wideband (UWB) modulations, the Impulse Radio (IR-UWB) and the Frequency Modulated UWB (FM-UWB) have interesting properties with regards to these requirements. First of all, since the spectrum is spread over a very wide band, the two UWB modulations are inherently more robust against frequency selective fading [3]. A significant attenuation at a specific frequency might prevent communication with a narrow-band radio, whereas it will only have a minor impact on a UWB radio. Second, FM-UWB radios are inherently resilient to narrow-band interference, achieving good performance without external components such as high-Q Surface Accoustic Wave (SAW) filters, and thus providing more potential for miniaturization. Large transmit spectrum also means that there is no need for precise frequency synthesis and no need for a Phase Locked Loop (PLL). This could allow for saving some power as the costly frequency dividers are not used, but also brings advantages in duty cycled networks where the transceiver is turned on and off periodically. Since a transceiver cannot turn on instantly, some energy will be wasted during the wake-up time. The wake-up time is usually limited by the wake-up time of the PLL due to its limited bandwidth and thus removing the PLL would mean reducing this energy overhead. The main downside of the UWB approach is that the large bandwidth requires more complex circuits that usually require more power; however, with all the benefits in mind, it remains an attractive choice for a WBAN.

The IEEE 802.15.6 standard was tailored to the needs of the WBAN [4]. It includes both the wideband and the narrow-band communication. Two UWB modulation schemes that became part of this standard are the Impulse Radio (IR-UWB) and Frequency Modulated UWB (FM-UWB). The IR-UWB uses narrow pulses that can be modulated using on-off (OOK), position (PPM), phase (PSK) or frequency (FSK) modulation (although only OOKs and PSKs are supported by the standard). It is intended for high data rates and can achieve a considerably low energy per bit. The price to be paid is the receiver complexity that comes from the stringent requirements for precise timing synchronization. The FM-UWB is a low complexity alternative that targets low and moderate data rates. Although it cannot achieve as low energy per bit as IR-UWB, it compensates with a smaller silicon area, easier implementation and lower peak power consumption. Thus, in applications that do not require very high data rates FM-UWB may usually be a better choice; however, in the IEEE 802.15.6 standard, it is only used as an optional mode.

**Figure 2:**
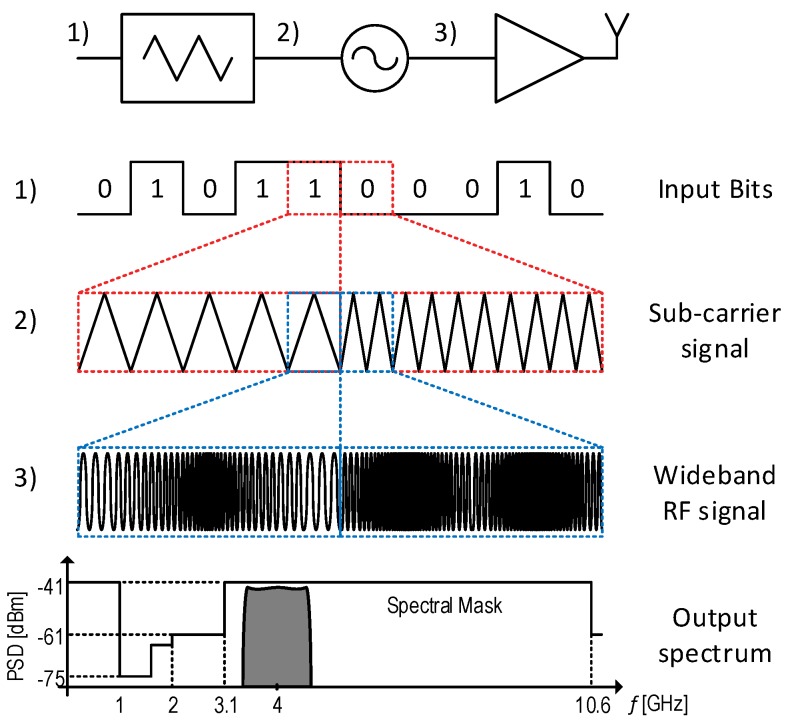
Principle of FM-UWB signal generation.

The standard FM-UWB signal is generated in two steps (Figure 2. First, the input bits are mapped to FSK symbols that use a triangular waveform (sub-carrier). Then, in the second step, these triangular waveforms are used to modulate the RF carrier and create the wideband signal. In this way, a flat spectrum with a steep spectral roll-off is obtained. The Physical (PHY) layer specifications in the IEEE 802.15.6 standard only define a 2-FSK sub-carrier modulation at a single mandatory RF channel and a single sub-carrier channel. As we intend to show in this paper, standard FM-UWB can easily be extended to higher data-rates and higher order FSK modulations without a significant increase in complexity and power consumption, which would result in a higher efficiency of the entire system. Since the IEEE 802.15.6 was not intended for a large number of devices, the potential of the FM-UWB was not fully exploited. The sub-carrier FDMA (SC-FDMA), which was demonstrated in [5,6,7], allows multiple nodes to communicate at the same time, which can bring a lot of benefits as the number of nodes in a network increases. Here, capabilities and limitations of multi-user communication are discussed, and the case with up to four users at the same RF channel is demonstrated. The concepts from [5] are further extended to provide a simple approximation for Bit Error Rate (BER) estimation. Furthermore, multi-channel transmission is proposed via a simple modification of the standard FM-UWB, which could allow simultaneous transmission to multiple nodes, as yet another technique that might find use in HD-WSNs.

The paper is organized as follows. The review and analysis of existing FM-UWB transmitters and receivers, pointing out the power reduction trends, are given in Section 2. In the following sections, the potential of FM-UWB beyond a simple 2-FSK sub-carrier modulation is demonstrated using one of the existing receivers. Single carrier communication is described in Section 3 and the SC-FDMA approach is explored in Section 4. The new multi-channel transmission is explained in Section 5, while Section 6 provides concluding remarks.

## 2. Review of FM-UWB Transceivers

### 2.1. FM-UWB Receivers

**Figure 3:**
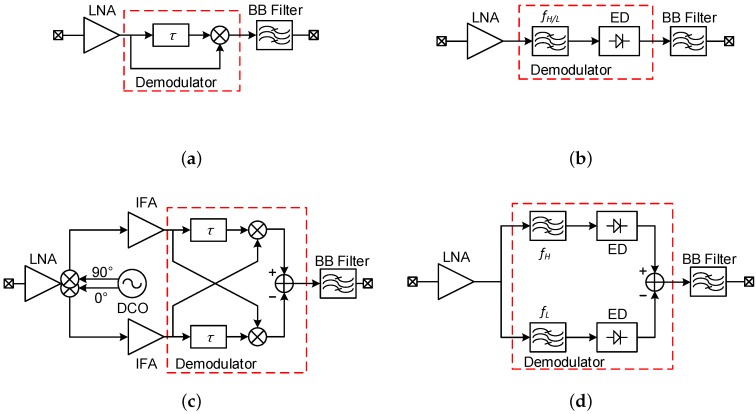
FM-UWB receiver architectures reported in literature: (**a**) delay-Line demodulator at RF; (**b**) regenerative demodulator; (**c**) baseband delay-Line demodulator and (**d**) dual-band-pass-filter demodulator (balanced frequency discriminator).

Different FM-UWB receiver implementations can be found in the literature. A simplified block diagram of each of the proposed architectures is shown in Figure 3. The wide-band FM demodulator based on a delay-line (DL) demodulator (Figure 3a) was initially proposed in [8]. Assuming that the delay is relatively small, the demodulated signal can be represented as [8]

(1) $$\begin{array}{c} V_{dem}={\left(-1\right)}^{(N+1)/2}\;\frac{A^{2}}{2}\sin\left(N\frac{\pi }{2\omega_{c}}\frac{d\phi (t)}{dt}\right),\;\mathrm{for}\;\tau =N\frac{\pi }{2\omega_{c}},\;N=1,3,5,..., \end{array}$$

where *A* is the amplitude of the demodulator input signal, $\omega_{c}$ is the carrier frequency and ϕ(t) is the useful frequency-modulated signal. The delay τ must be chosen to be an odd multiple of the carrier frequency in order to cancel out the offset in the demodulated signal [8]. The choice of *N* is a trade-off between the conversion gain and the bandwidth of the demodulator. Lower value of *N* leads to lower conversion gain, but also increases the useful frequency range. In practice, it will be very difficult to achieve high precision of the delay (due process variation), and so a small offset in the demodulated signal will always be present. However, since the transmitted signal is at least 500 MHz wide, this small offset should not have a major impact on the receiver performance. The first fully integrated FM-UWB receiver based on a DL demodulator was described in [6]. It achieves a sensitivity of −88 dBm while consuming 9.4 mW. The demodulator itself consumes around 5.8 mW, while the Low Noise Amplifier (LNA) consumes 3.6 mW.

**Figure 4:**
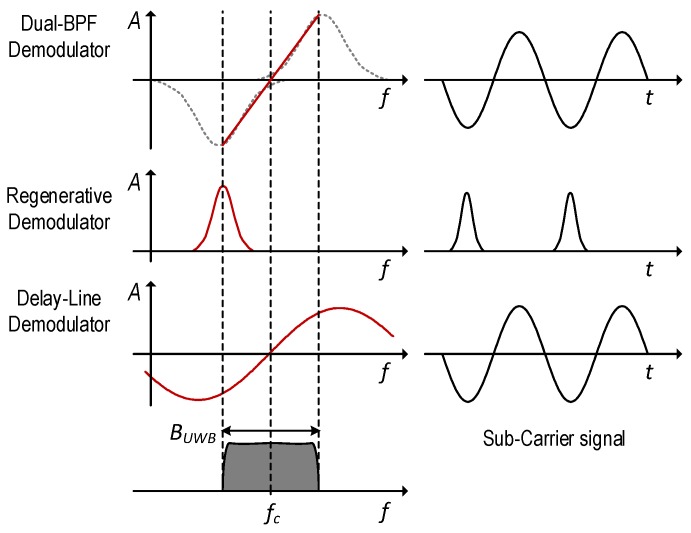
Frequency-to-amplitude conversion characteristic of different FM demodulators found in literature.

An LNA that provides high gain across a large bandwidth inevitably requires more power compared to a narrowband LNA. In order to reduce the power consumption, a narrow-band regenerative receiver was proposed in [9]. This approach allowed to preserve high gain and relatively good noise figure, while minimizing the power consumption. The high-Q band-pass filter behaves as a frequency discriminator that converts the input FM signal into an AM signal, which is then converted to Intermediate Frequency (IF) using an envelope detector. Due to the high-Q factor of the filter that results in a very nonlinear Frequency Modulation to Amplitude Modulation (FM-AM) conversion characteristic, the demodulated signal will be a train of pulses whose frequency corresponds to the sub-carrier frequency (Figure 4). The receiver from [9] consumes 2.2 mW while achieving –84 dBm sensitivity. A later implementation presented in [10] introduced several improvements at the circuit level (most notably current reuse among several blocks), which resulted in power consumption of only 560 µW and a slight reduction of sensitivity. Although the regenerative receiver achieved significant power savings, there are some downsides to this architecture. Narrow-band interferer rejection mostly relies on the high-Q input filtering. If the interferer falls inside the pass-band, it could easily saturate the stages following the LNA and prevent reception. The second downside comes from the nonlinear FM-AM conversion. If several FM-UWB signals were to occupy the same RF band, the weaker signals would be attenuated in the nonlinear conversion process, which would prevent correct demodulation. This is known as the capture effect [11] and limits the regenerative receiver to cases where only one FM-UWB signal is transmitted in a given RF band.

In an attempt to improve the linearity of the regenerative demodulator, a modified architecture was proposed in [12]. Instead of using just one band-pass filter, a second branch was added (Figure 3d), resulting in a Dual Band-Pass Filter (DBPF) demodulator, otherwise known as a balanced frequency discriminator. The two filters are tuned to the two extreme frequencies in the FM-UWB signal spectrum, and are followed by the two envelope detectors that are removing the RF carrier from the signal. The difference of the two IF signals finally gives the demodulated sub-carrier. The equivalent linearized characteristic is shown in Figure 4. Compared to the original regenerative receiver, the Q-factor of the two filters can be lowered, which allows some power savings per filter, but two filters are now needed, as well as a wide-band LNA, which is again the dominant source of power consumption. The two architectures perfectly illustrate the trade-off between linearity and power consumption in FM-UWB receivers. The implementation from [12] consumed 3.8 mW, and achieved −78 dBm of sensitivity. The same architecture was reused in [13] for demodulation of a Chirp-UWB signal, where symbol-level duty-cycling of the receiver was used to bring down the average power consumption to 0.6 mW. The DBPF receiver exhibits better narrow-band interferer rejection compared to a standard regenerative receiver and should perform better in scenarios with multiple FM-UWB users, although such capability was not confirmed by measurements.

In all of the mentioned examples, the most power hungry blocks are the ones that operate at high frequencies. Typically, this is the LNA, but also the demodulator in the case of the DL architecture. Assuming that the oscillator can be made sufficiently low power, the input signal could be downconverted to IF first and then amplified and processed, allowing for achieving the same gain at a lower cost in terms of power. This approach was first proposed in [14] for a narrow-band OOK receiver. Similar architecture was applied to the case of FM-UWB in [7], it uses an active mixer-first topology to downconvert the signal and then amplifies it using IF amplifiers. In order to achieve low power consumption, a ring oscillator is used to generate the Local Oscillator (LO) signal. Increased phase noise and frequency drift due to Process-Voltage-Temperature (PVT) variations are not a big concern owing to the large FM-UWB signal bandwidth. The demodulator used in this receiver is again a DL demodulator. Since in this case it operates at baseband instead of RF, quadrature signals are needed for correct demodulation. Compared to the RF-DL demodulator, the delay τ no longer depends on the carrier frequency, and does not need to be set precisely. Furthermore, by moving the demodulator from RF to baseband, its power consumption can be reduced by more than two orders of magnitude (from 5.8 mW to 26 µW). The obvious downside of any mixer-first receiver is the increased noise figure that results in lower sensitivity. The receiver from [7] achieved –70 dBm of sensitivity while consuming 420 µW and is so far the lowest power FM-UWB receiver. Even though there is a penalty when it comes to sensitivity, this receiver can still distinguish multiple FM-UWB signals, and provide SC-FDMA, which is a useful property in dense WBANs. In fact, the aforementioned receiver was designed specifically for communication between the nodes on a prosthetic limb [2] and trades sensitivity for power consumption and FDMA capability. In cases where higher path losses are expected and the number of nodes is smaller, such as in [15], a different receiver would be more optimal.

A performance summary of different FM-UWB receivers is given in Table 1. Each of the proposed architectures has its own advantages and disadvantages. Receiver from [6] generally has the best performance but is also the most power hungry. Regenerative receiver can provide a very low power consumption while maintaining good sensitivity, but at the cost of linearity. Finally, the DL based receiver from [7] sacrifices sensitivity but achieves the lowest power and preserves FM-AM linearity.

### 2.2. FM-UWB Transmitters

Unlike the FM-UWB receivers, the architecture of the FM-UWB transmitters has remained unchanged over the past several years. Considering its simplicity (Figure 2), it is clear that there is not a lot of potential for improvement on the architectural level. In fact, the reduction of power on the transmitter side is mainly a result of improvements on the circuit level. Every FM-UWB transmitter consists of three blocks, the sub-carrier generator, the Voltage Controlled Oscillator (VCO) (sometimes as a part of a PLL or a Frequency Locked Loop (FLL)) and a power amplifier (PA).

The sub-carrier generator generates the triangular waveform that is used to drive the VCO. As the sub-carrier frequencies are rather low (typically 1–2 MHz), this block does not contribute significantly to the overall transmitter consumption. One way to implement it is a Direct Digital Synthesis (DDS) as described in [20]. The advantages of digital implementation are the simple and precise frequency control without the need for calibration. The drawback of the fully digital approach becomes apparent at higher data rates, where higher sub-carrier frequencies are needed. In [21], 51 MHz sub-carrier frequency is used. Since roughly 20 points per period are needed to generate a reliable sub-carrier waveform, a DDS would need to operate at a clock speed of more than 1 GHz, which would be difficult to implement and would consume a significant amount of power. Instead, a relaxation oscillator is used within a PLL, as a more optimal solution. Another interesting approach that leads to very low power consumption is a free-running relaxation oscillator, which is periodically calibrated using an FLL [22]. Digital frequency control is provided through a capacitor bank; however, this approach is usually not precise enough if multiple sub-carrier channels are to be used.

The two parts of the FM-UWB transmitter that essentially determine its power consumption are the VCO and the PA. In the case where transmitted power is 10 dBm or more, the transmitter efficiency was dominated by the PA, however at lower output powers, such as, at −10 dBm, the contribution of the VCO becomes quite significant. In some of the earlier implementations, the RF carrier was synthesized using an LC VCO within a PLL [21,23]. To decrease power, the frequency synthesizer is duty cycled, making the frequency dividers active for only 10% of the time. Although this allowed some savings, the power consumption was still on the order of 10 mW. A significant improvement was made when the LC oscillator was replaced with a ring oscillator [22,24]. This was possible owing to the loose phase noise constraints of the FM-UWB modulation. Additionally, instead of the quasi-continuous PLL, an FLL calibration loop was used [22]. Since the FM-UWB spectrum is very wide, the center frequency can deviate slightly without a major impact on performance and it does not need to be monitored continuously. Therefore, once calibrated, the VCO can operate in a free running manner until temperature or some other external factor causes a significant frequency shift. Since these external processes are usually slow, calibration only needs to be done once in a few hours or days, which makes the average power consumption of such an FLL practically negligible. The described approach led to the first sub-milliwatt FM-UWB transmitter [22]. The next step in reducing the VCO consumption was reducing the frequency of oscillation. Since an *N*-stage ring oscillator produces *N* equally spaced phases, these phases can be combined to produce a frequency that is *N* times higher [25]. It is then possible to use a ring oscillator that works at a frequency that is *N* times lower than the carrier center frequency. The approach was demonstrated in [25] and used for the FM-UWB transmitter in [10] to reduce the power consumption down to 0.63 mW.

Even though the VCO cannot be neglected, the PA remains the most power-hungry block in the system. The key to further reducing the power consumption of an FM-UWB transmitter is an efficient power amplifier; however, the design of an integrated PA for such a low power and wide band poses a number of challenges. In standard narrow-band applications targeting relatively high output power, the most efficient approach is to use a switching PA such as class D or E. However, due to the high driving requirements, lack of voltage gain and difficulties in implementing a wideband matching network, switching PAs leads to sub-optimal solution. The linear power amplifiers, classes A, AB, B and C, generally do not achieve as high efficiency, but the driving requirement is also lower. Moving from class A to class C operation, the maximum attainable efficiency increases, but the power gain decreases and larger driving signal is necessary, thus shifting the burden from the PA to the driver. A good compromise is the class AB that attains decent efficiency and does not need a rail-to-rail input signal. In fact, all of the transmitters reported in [10,22,24], which achieve the lowest power consumption so far, use a complementary class AB power amplifier. Finally, by applying the current reuse technique, as demonstrated in [10], where the PA and the driver share the same current, efficiency can be further improved. This led to a record breaking low power FM-UWB transmitter as can be seen in Table 2.

### 2.3. Summary

**Figure 5:**
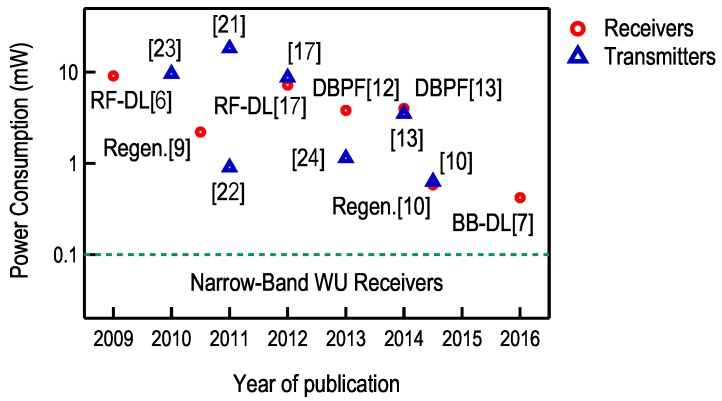
FM-UWB transmitters and receivers, evolution of power consumption. Type of demodulator used in each receiver is indicated on the graph.

The above sections described the most important power reduction techniques applied in the FM-UWB transceivers. These techniques, combined with technology scaling, led to sub-milliwatt power levels in today’s implementations. The evolution of power consumption over the past eight years is illustrated in Figure 5 for both transmitters and receivers.

Although there has been a decrease by a factor of 20, the narrow-band receivers still have the edge, at least when it comes to power consumption. Proposed wake-up receivers found in literature consume from 100 µW [26] all the way down to 100 nW [27]. FM-UWB can hardly compete with such low levels, a simple consequence of the fact that wide-band circuits require more power to achieve the same performance in terms of gain and noise figure. On the other hand, the FM-UWB brings higher resilience to interferers without off-chip components such as SAW filters, better performance in frequency selective channels and more potential for miniaturization. All of these are very favorable capabilities that could assure a place for FM-UWB in short-range applications such as Body Area Networks.

## 3. Single-User FM-UWB Communication

Although different FM-UWB receiver architectures have been explored, most of them (with the exception of [13]) focused on standard 2-FSK sub-carrier modulation, targeting data rates below 100 kb/s. Transmitters proposing higher data rates (1 Mb/s) and more complex M-FSK modulations have been implemented, but the full communication has never been demonstrated with one of the existing receivers. Here, the receiver from [7,19] was used to demonstrate the feasibility of communication at higher data rates and using M-FSK without any modification to the wideband FM demodulator. In fact, any of the architectures shown in Figure 3 could be used to accommodate different sub-carrier modulations. Minor modifications would be needed only in the baseband circuitry. As the power consumption of the baseband part is typically negligible compared to the RF part, increasing communication speed up to a certain point would only result in a small increase of the overall consumption. It is therefore expected that higher speed leads to a higher energy efficiency of the entire system.

**Figure 6:**
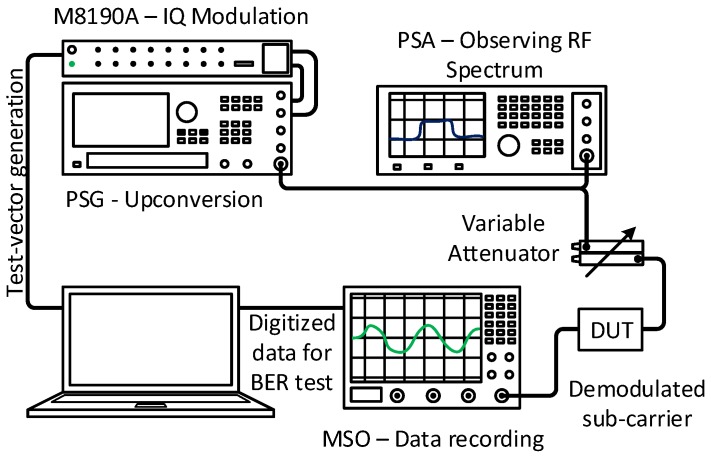
Test setup used for measuring performance of FM-UWB receiver with different sub-carrier modulations.

The transmitted signal was generated using two signal generators. A 12 GS/s, 12-bit arbitrary waveform generator M8190A provides the quadrature baseband FM-UWB signal. This generator allows generation of any signal up to 4 GHz of baseband bandwidth and was used to create different transmission scenarios (including the multi-user communication). The quadrature signal is then upconverted to 4 GHz using the PSG signal generator. The whole test setup is shown in Figure 6. Signal power at the receiver is controlled using a variable attenuator. The output data from the FM-UWB receiver is digitized and then processed off-line to obtain the Bit Error Rate (BER).

**Figure 7:**
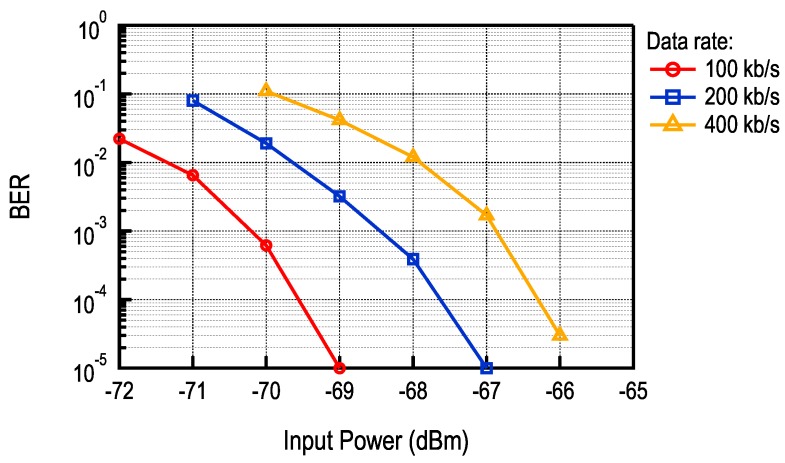
BER for different data rates.

The BER curves for different data rates are shown in Figure 7. In all cases, orthogonal frequencies are used with a modulation index of 0.5. As expected, the sensitivity of the receiver decreases due to increased bandwidth of the sub-carrier signal. Nevertheless, if the distance between nodes is small enough and the channel is sufficiently good, higher communication speeds can be used to decrease latency in the network. In this case, the limitation to data rate comes from the filter following the FM demodulator, which was designed for sub-carrier frequencies from 1–2.5 MHz.

**Figure 8:**
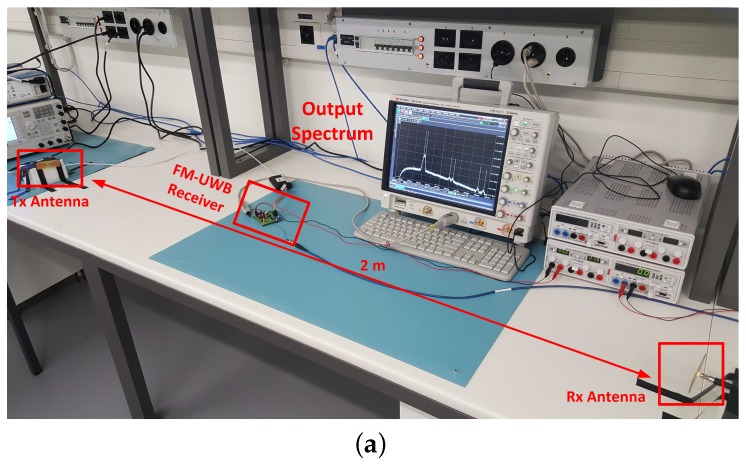

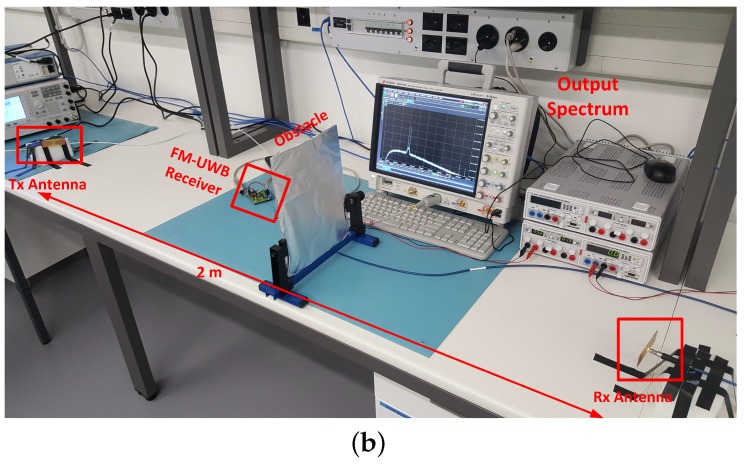
Over the air measurement (**a**) with direct line of sight and (**b**) without the direct line of sight. Signal is transmitted at −15 dBm, distance between the transmit and receive antenna is roughly 2 m, 100 kb/s, and 2-FSK modulation is used.

Figure 8 shows the test setup that demonstrates over the air communication. The distance between the two antennas is approximately 2 m, roughly corresponding to the distances that are targeted for short range communications in a WBAN (although still simplistic compared to a real use-case scenario). The signal is transmitted at −15 dBm, somewhat lower than the allowed power in the UWB frequency range. Oscilloscope screen shows the spectrum of the demodulated signal (using the FFT). Two cases are presented. In the first, there is a line of sight propagation between the transmit and the receive antenna, while, in the second, an obstacle is placed between the two antennas. The degradation of the link quality can be observed on the oscilloscope screen once the obstacle is in place; however, it is still possible to establish communication. In the first case, BER is below $10^{-5}$, while, in the second, it increases to $3.1\times 10^{-4}$. In both cases, 100 kb/s 2-FSK modulation is used. The two examples are shown; however, the BER will vary as the location of the obstacle changes, as well as the surroundings in general.

**Figure 9:**
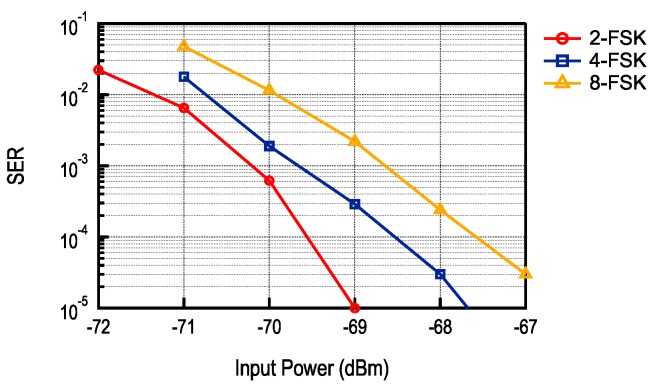
SER for 2-FSK, 4-FSK and 8-FSK sub-carrier modulation.

The example in Figure 9 shows performance, in terms of Symbol Error Rate (SER), for different M-FSK modulations. With the implemented receiver baseband bandwidth, up to 8-FSK signal can be received at 100 ksym/s. In general, M-FSK offers better sensitivity for the same data rate; however, it also requires a more complex M-FSK demodulator. The selection of the most efficient modulation then depends on the targeted application.

**Figure 10:**
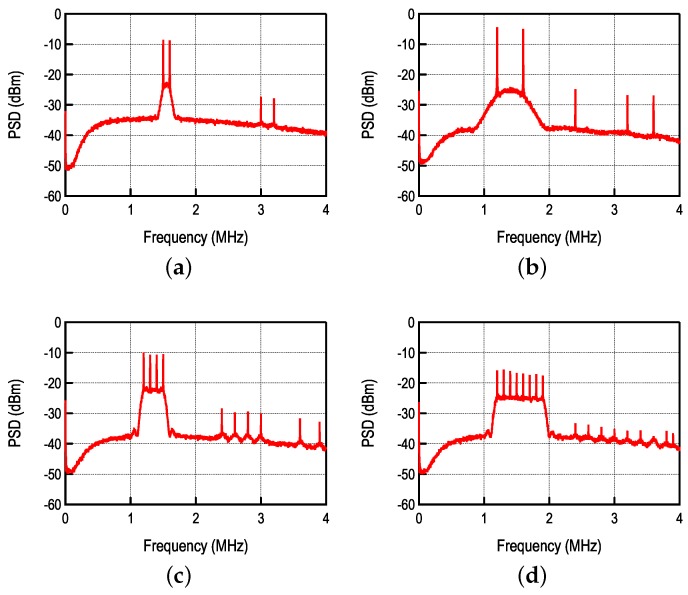
Demodulated spectrum for different symbol rates and different sub-carrier modulation complexity: (**a**) 100 kb/s, 2-FSK modulation; (**b**) 400 kb/s, 2-FSK modulation; (**c**) 100 ksym/s, 4-FSK modulation and (**d**) 100 ksym/s 8-FSK modulation.

The spectrum at the output of the wideband demodulator is shown in Figure 10 for some of the used modulations. If only a single transmitter is using the given RF band, there is no reason not to use the entire available sub-carrier band; however, if multiple nodes need to transmit, the bandwidth can be reduced to provide enough room for additional channels. This potential for configurability is one of the strong points of FM-UWB.

## 4. Multi-User FM-UWB Communication

Frequency Division Multiple Access is a concept that has been exploited for a long time. Compared to wireless communication on a single frequency channel, which requires some form of Time Division Multiple Access (TDMA), it enables us to speed up data transfer from multiple nodes in a network and therefore decrease latency and improve response time to environment changes. The FDMA not only provides multiple access for devices in a WBAN but also offers the potential to separate WBAN traffic by service type and priority, which facilitates tailoring to the needs of the application. For example, a real-time application can operate on a separate channel, while bursty low rate sensor data is aggregated on another channel.

When it comes to FM-UWB, there are two ways to implement FDMA. First, the straightforward way is to use different UWB channels at RF. Although it may seem simple, it requires a fairly large bandwidth in the receiver front-end and may lead to significantly larger power consumption. Nearly all of the receivers reported in literature support only a single RF UWB channel, thus showing the impracticality of the aforementioned approach. The second way is to use multiple sub-carrier frequencies (SC-FDMA), while only transmitting on a single RF channel. The main advantage of SC-FDMA is that the modifications are primarily needed in the low frequency part of the receiver. As the power overhead remains relatively low, this approach is more appealing for applications such as body area networks and will be discussed in this section.

**Figure 11:**
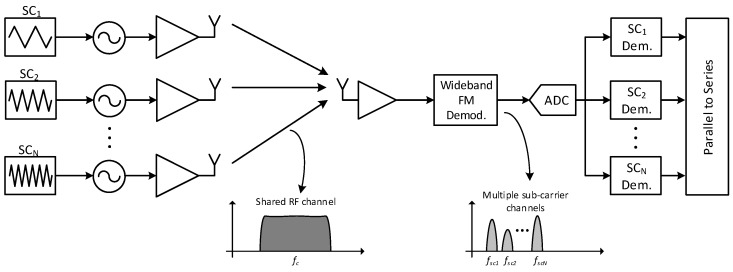
Multiple transmitters sharing the same RF band.

The SC-FDMA was proposed in [8] and further studied in [5]. The main principle is shown in Figure 11, where multiple transmitters are using the same RF band, but can be distinguished due to the fact that they use different sub-carrier frequencies. In order to perform successful demodulation, the wideband FM demodulator must provide sufficient linearity in order to avoid distortion and FM capture effect. In that regard, the DL demodulator is the best candidate, and, so far, the only one that has demonstrated such capability [6,7].

In order to allow simultaneous demodulation, the baseband FSK demodulator must be replicated for each sub-channel. A fully analog approach was described in [8,28], and digital sub-carrier processing is proposed here. Firstly, the dynamic power consumption of digital circuits decreases with technology scaling and is likely to provide a lower power solution in the future. Furthermore, Analog to Digital Converters (ADCs) are now capable of achieving very low power operation, such as the 10 MS/s, 8-bit ADC implemented in [29] that consumes only 26 µW. Secondly, the digital approach offers easier configurability, meaning that the same receiver could be used to either receive data from multiple users at a lower speed, or to increase speed and efficiency for single user communication.

**Figure 12:**
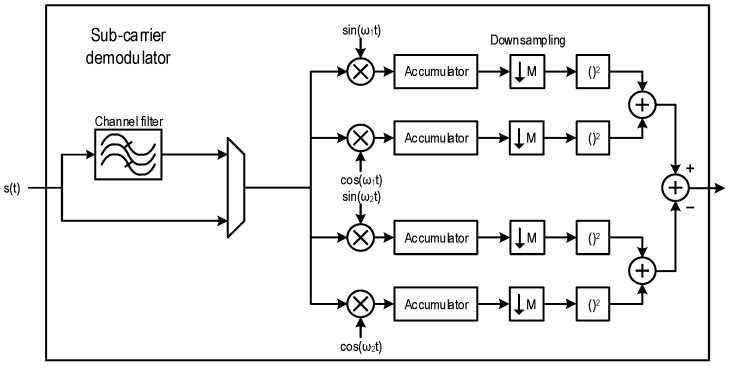
Block diagram of a single sub-channel demodulator.

A block diagram of an FSK demodulator based on correlators for processing a single sub-channel is shown in Figure 12. The proposed demodulator only illustrates the principle, and different simplifications can be used to optimize power consumption. For example, instead of using a sine wave, a square wave $sgn(\sin\left(\omega_{sc}\right)t)$ could be used, which would allow for removing multipliers and reducing complexity. The channel filter, which is the most demanding block here, only needs to be used if significantly stronger adjacent sub-channels are present; otherwise, it can be switched off to save power.

Effectively, replicated sub-channel demodulators constitute a correlator bank. In cases where only one transmitter is used, this correlator bank could be exploited to demodulate the M-FSK signal, allowing higher speed in a single sub-channel as mentioned earlier. Different variations could also be supported, trading the number of channels with the data rate per sub-channel. The given example highlights the potential and versatility of the digital approach. The same FM-UWB receiver can be used to adapt to different conditions in the network, maximizing system efficiency and providing more room for improvement at the protocol level.

**Figure 13:**
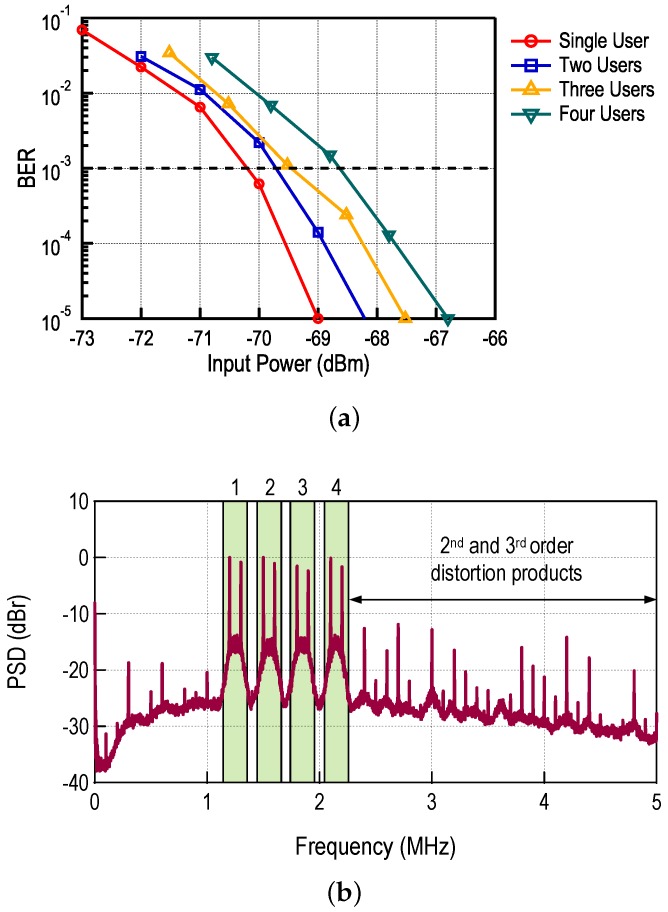
SC-FDMA with equal power transmitters, (**a**) BER curves for different number of users and (**b**) spectrum of the demodulated signal for the case of four sub-channels.

To measure the performance in the presence of multiple FM-UWB signals the same setup from Figure 6 was used. The measured BER performance is presented in Figure 13a for a number of users varying from 1 to 4. The data rate on each sub-channel is 100 kb/s resulting in 200 kHz bandwidth, with 100 kHz separation between the channels. Orthogonal 2-FSK modulation without pulse shaping is used in all cases. The output spectrum is shown in Figure 13b.

The multi-user interference and noise performance of the FM-UWB were studied in [5,8] for the case of a delay-line demodulator. However, the results from [5] only addressed limits due to inter-user interference and did not take noise into account. Inter-user interference becomes a dominant factor only at large variations in power between the sub-channels, and it is of interest to assess the BER performance and sensitivity degradation in other cases. Following the same reasoning used by Gerrits in [5,8], assuming that the delay is sufficiently small and the noise autocorrelation function $R_{n}\left(\tau \right)=0$ the demodulator output voltage, in the case of two users, becomes

(2) $$\begin{array}{ccc} V_{o,2} & = & k{\left(V_{s1}+V_{s2}+V_{n}\right)}^{2} \end{array}$$

(3) $$\begin{array}{cc} = & k\left[V_{s1}^{2}+V_{s2}^{2}+V_{n}^{2}+2V_{s1}V_{s2}+2V_{s1}V_{n}+2V_{s2}V_{n}\right], \end{array}$$

where *k* is the constant of the demodulator that has a dimension $V^{-1}$. Since this constant does not affect the output Signal to Noise Ratio (SNR), it can be dropped from further consideration. The factors $V_{s1}^{2}$ and $V_{s2}^{2}$ are the two demodulated sub-channels. The useful signal power on channel *i* is then proportional to

(4) $$\begin{array}{c} S_{out,i}\propto V_{si}^{4}\propto S_{i}^{2}, \end{array}$$

where $S_{i}$ is the input signal power on channel *i*. The other terms in Equation (3) correspond to cross-products of signal and noise ($V_{si}V_{n}$) and the two input signals ($V_{si}V_{sj}$), and noise squared ($V_{n}^{2}$) [8]. We can then write:

(5) $$\begin{array}{ccc} 2\sqrt{S_{i}S_{j}} & \propto & 2V_{si}V_{sj}, \end{array}$$

(6) $$\begin{array}{ccc} 2\sqrt{S_{i}N} & \propto & 2V_{si}V_{n}, \end{array}$$

(7) $$\begin{array}{ccc} N & \propto & V_{n}^{2}, \end{array}$$

where *N* is the input noise power. The total output noise power, as a function of input signal power and noise power is then given by

(8) $$\begin{array}{c} N_{out}=N^{2}+4S_{1}N+4S_{2}N+4S_{1}S_{2}. \end{array}$$

Finally, the SNR at the demodulator output is given by

(9) $$\begin{array}{c} SNR_{out,2}=\frac{B_{RF}}{B_{sub}}\frac{S_{1}^{2}}{N^{2}+4S_{1}N+4S_{2}N+4S_{1}S_{2}}, \end{array}$$

where factor $B_{RF}/B_{sub}$ is the processing gain coming from the spread spectrum FM-UWB technique [8]. Let us now assume that the two sub-carrier channels have equal power $S_{1}=S_{2}$. The output SNR becomes

(10) $$\begin{array}{c} SNR_{out,2}=\frac{B_{RF}}{B_{sub}}\frac{S_{1}^{2}}{N^{2}+8S_{1}N+4S_{1}S_{2}}=\frac{B_{sub}}{B_{RF}}\frac{SNR_{in}^{2}}{1+8SNR_{in}+4SNR_{in}^{2}}. \end{array}$$

This expression can be compared to the SNR in the case of a single user FM-UWB:

(11) $$\begin{array}{c} SNR_{out,su}=\frac{SNR^{2}}{1+4SNR_{in}}. \end{array}$$

It should be noted that the output SNR is a non-linear function of the input SNR, which is a consequence of the square characteristic of the demodulator. Although the derived SNR expressions are a rather coarse approximation, they provide some intuitive insight into the performance limitations. First, the achievable BER is now limited by the inter-user interference ($4S_{1}S_{2}$), which fundamentally limits the number of users or the maximum difference in powers between the two users. This factor, however, remains relatively small compared to noise at low SNR values. What can be noted in this region is that the “noise floor” has now increased due to cross-modulation of several input signals with noise ($8S_{1}N$). In case a small number of channels is used, and the power difference between channels is small, this factor will dominantly determine the sensitivity degradation. The same derivation can be applied to the cases with three and four users:

(12) $$\begin{array}{ccc} SNR_{out,3u} & = & \frac{B_{RF}}{B_{sub}}\frac{SNR_{in}^{2}}{1+12SNR_{in}+12SNR_{in}^{2}}, \end{array}$$

(13) $$\begin{array}{ccc} SNR_{out,4u} & = & \frac{B_{RF}}{B_{sub}}\frac{SNR_{in}^{2}}{1+16SNR_{in}+24SNR_{in}^{2}}. \end{array}$$

**Figure 14:**
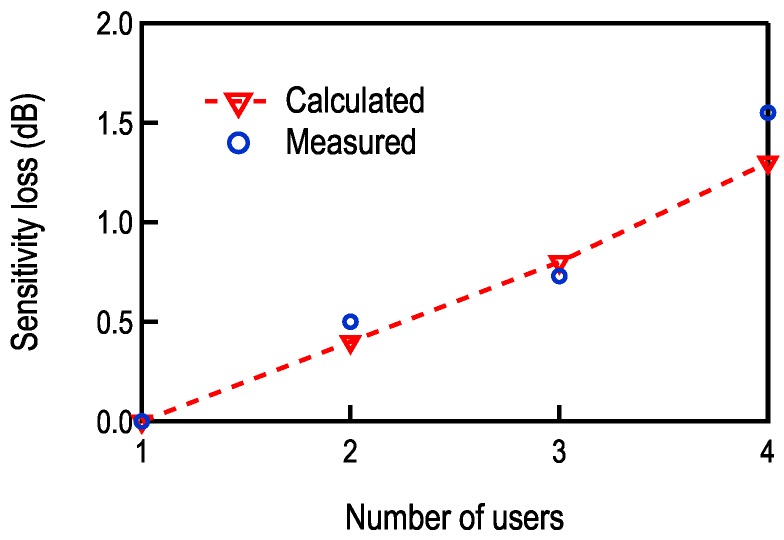
Calculation and measurement of sensitivity degradation due to the presence of multiple users.

The probability of error can then be calculated using the standard formula for the orthogonal 2-FSK modulation:

(14) $$\begin{array}{c} P_{e}=Q\left(\sqrt{\frac{E_{b}}{N_{0}}}\right),\;\frac{E_{b}}{N_{0}}=\frac{B_{sub}}{R}SNR_{out}, \end{array}$$

where *R* is the data rate. The presented calculation can be used to estimate the sensitivity loss due to the presence of multiple users, sensitivity defined as BER = $10^{-3}$. The calculation is compared to measurements in Figure 14. A slight deviation from calculation can be noticed in the case with four users. The likely cause is the non-linearity of the baseband circuits. The third order intermodulation product (IM3) from channels 3 and 4 falls directly on top of channel 2 (used for measuring the BER), which degrades the equivalent SNR:

### Limitations to Multi-User Communication

The first fundamental limitation comes from the inter-user interference, which limits the minimum achievable BER. In the high SNR region, the noise can be neglected and Equation (10) can be rewritten as [5]

(15) $$\begin{array}{c} SNR_{out}=\frac{B_{RF}}{B_{sub}}\;\frac{S_{1}}{4S_{2}}. \end{array}$$

Assuming orthogonal FSK, 500 MHz RF bandwidth and 100 kHz data rate, a 21 dB stronger RF signal using sub-channel 2 will limit the BER on channel to $10^{-3}$. In other words, the desired BER imposes the maximum difference in power of different channels that can be tolerated. In the same way, inter-user interference will limit the number of equal power channels that can be used. If multi-user performance is to be improved, the only remaining degree of freedom is to increase the processing gain. It should be noted that, due to the quadratic characteristic of the demodulator, 21 dB difference of power at RF corresponds to 42 dB difference in the power of the sub-carriers. This shows that it is actually more likely that the difference in channel power will be limited by the dynamic range of the receiver than the inter-user interference. Typically, there is a trade-off between power and dynamic range in amplifiers, so the capability to handle larger sub-channel power difference will come at the price of an increased power consumption. One way to avoid this is to regulate transmit power in order to avoid the need for high dynamic range and hence higher power (as it is done in CDMA).

**Figure 15:**
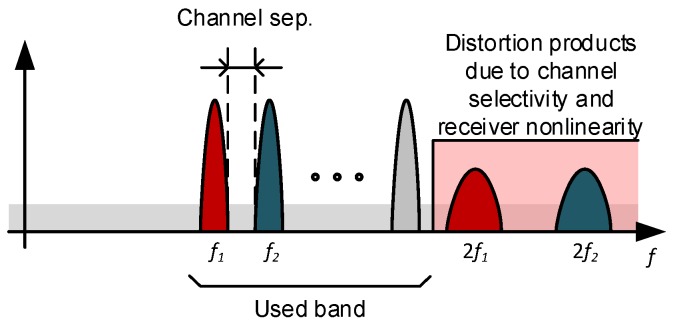
FSK sub-channel frequency allocation and limits due to distortion.

The second limitation comes from distortion. Any frequency selectivity present in the channel due to multipath propagation or non-linear demodulator characteristic will cause AM to appear on top of the FM-UWB signal. These amplitude variations transform into harmonic distortion in the process of demodulation and pollute the spectrum. If the minimum sub-carrier frequency is $f_{sub}$, then the spectrum at $2f_{sub}$ and above will contain significant components due to unwanted AM as illustrated in Figure 15. Placing a channel directly on top of these frequencies would result in significant BER degradation, and, for this reason, wouldn’t be practical. The sub-carrier band is therefore limited to one octave. If a larger band is required, the solution would be to move to higher sub-carrier frequencies. In the case shown here, frequencies from 1.1 MHz to 2.2 MHz are allocated to sub-carrier channels.

The third limitation comes from the Adjacent Channel Leakage Ratio (ACLR). The spectrum of the FSK modulated sub-carrier spreads beyond the sub-channel bandwidth. If one sub-carrier is significantly stronger than others, spectral leakage might prevent correct detection on adjacent channels. To prevent this, channels can be separated, but then the total number of available sub-channels decreases. Otherwise, pulse shaping can be applied on the transmitter side to reduce the ACLR, but at the cost of increased complexity. In the case presented here, pulse shaping is not used, and 100 kHz channel separation was sufficient, but, in general, different parameters of the FM-UWB system can be set to conform to the specific needs of the application and provide the optimal solution.

## 5. Multi-Channel Transmission Using FM-UWB

**Figure 16:**
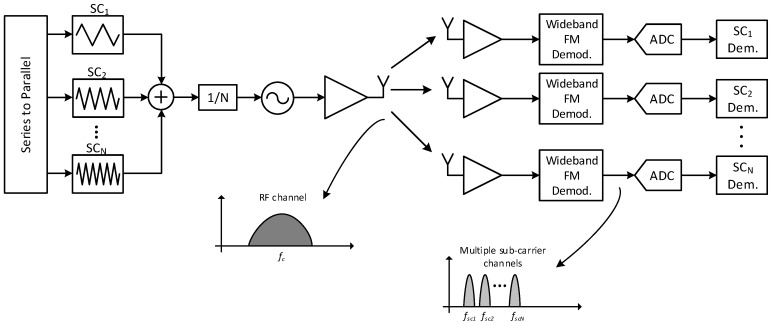
Single transmitter broadcasting on multiple sub-channels.

If a small modification is introduced, the FM-UWB can be used to transmit on multiple sub-carrier channels simultaneously. Instead of driving the VCO using a single sub-carrier signal, a sum of multiple sub-carrier signals could be used (Figure 16). In this way, a single transmitter can broadcast different messages to different receivers at the same time—a capability that could be useful if, for example, multiple actuators needed to be driven synchronously. Regarding power consumption, the proposed technique would only result in a minor increase. Since the most power-hungry blocks are the VCO and the power amplifier, increasing the number of baseband sub-carrier generators would not cause a significant overhead. As an example in [10], the VCO and the PA consume 92% of the overall power, while the SC generator consumes around 2%. Using four SC generators would thus result in an overhead of less than 10% in terms of power consumption, assuming minor modifications in other baseband blocks. In many ways, the proposed scheme is similar to having multiple parallel transmitters; however, there are some differences.

**Figure 17:**
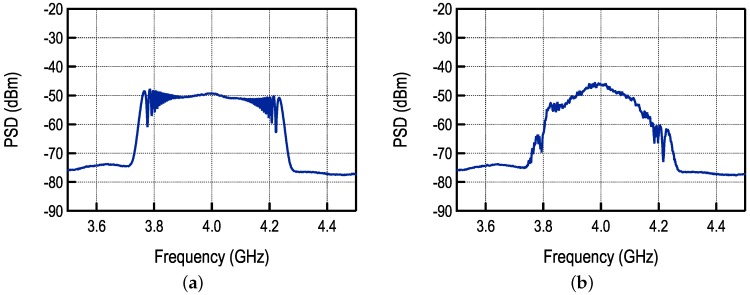
Spectrum of the transmitted signal in the case of (**a**) standard FM-UWB signal and (**b**) FM-UWB signal with multiple sub-carrier channels.

The FM-UWB spectrum is relatively flat and has a steep spectral roll-off that is a consequence of the triangular sub-carrier signal. Since several sub-carrier signals are summed here, the output spectrum will no longer be flat. In fact, the exact shape of the spectrum will depend on the number of sub-carriers and their initial phases. Furthermore, the sum of sub-carriers must be scaled in order to maintain the RF bandwidth. If *N* sub-carrier signals are combined, the sum must be divided by *N*. The output spectrum resulting from the addition of three sub-carrier channels is shown and compared to a standard FM-UWB spectrum in Figure 17. The scaling of sub-carriers is essentially equivalent to reducing the spreading factor of each sub-carrier. As a consequence, the power of each demodulated sub-carrier will be lower compared to the single sub-carrier case, which will result in worse sensitivity as the number of sub-channels increases. A degree of freedom that can be exploited here is using different scaling factors for different sub-channels. This is equivalent to allocating different power to sub-channels. If the information is transmitted to nodes at different distances, more power can be allocated to channels intended to more distant nodes in order to improve the link quality.

**Figure 18:**
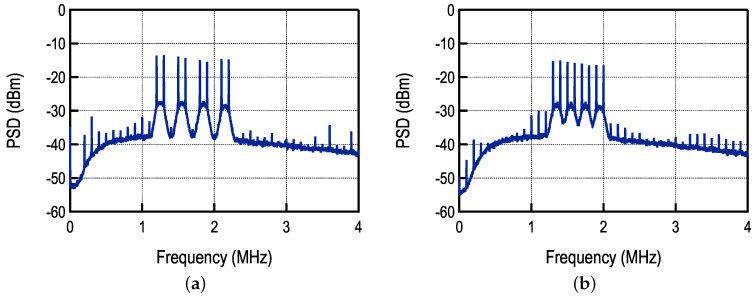
Spectrum of the demodulated signal (**a**) with and (**b**) without spacing between sub-carrier channels.

One important difference between SC-FDMA with multiple transmitters and a single transmitter is that, in the latter, sub-channels are perfectly synchronized. For multiple transmitters, even if the orthogonal frequencies are used for different sub-channels, the orthogonality is preserved only if symbols are perfectly synchronized. Since it is practically impossible to synchronize multiple transmitters, sub-channels must be separated and a channel filter is required. In the case of a single transmitter, the sub-channels remain orthogonal, so there is no need for separation, which allows for increasing the number of available channels. As long as the orthogonality is maintained, this separation will not influence the BER. In that regard, the proposed FDMA scheme is similar to the Orthogonal Frequency Division Multiplexing (OFDM) combined with the FM-UWB spread spectrum technique. The spectrum of the demodulated signal with and without separation is shown in Figure 18.

**Figure 19:**
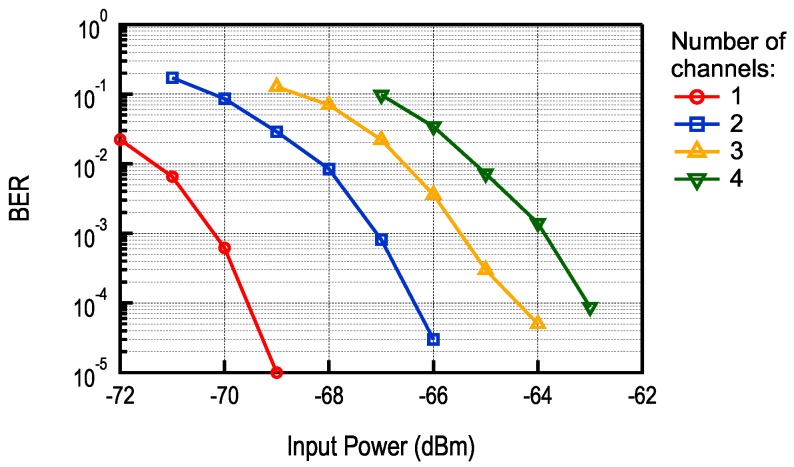
BER for a single transmitter broadcasting on multiple sub-channels.

The BER curves for different number of sub-channels are shown in Figure 19. Compared to the case with multiple transmitters, a significant sensitivity loss can be observed. This is a consequence of scaling since the SNR of a single channel is proportional to $1/N^{2}$, where *N* is the number of sub-channels. Nevertheless, for short range applications, where distance between nodes does not exceed several meters, such as BAN, the proposed scheme could still be used and could be of particular interest when a large number of nodes are present.

## 6. Conclusions

The FM-UWB has been recognized as a technique that finds use in applications such as WBANs due its very low power consumption, potential for miniaturization and low complexity. Although FM-UWB receivers can never achieve as low power as the narrow-band wake-up receivers, they compensate with small size, robustness in multi-path channels and resilience against interferers. The FM-UWBs became part of the IEEE 802.15.6 standard; however, the standard specifications limit the use of FM-UWB to the very basic case of the 2-FSK sub-carrier modulation. If a small increase in the complexity of the receiver can be afforded, FM-UWB can achieve higher data rates, either by simply increasing the speed and the sub-carrier frequency or by using higher order modulations such as 4-FSK and 8-FSK. The FM-UWB also provides support for communication on multiple sub-carrier frequencies through SC-FDMA and, with a minor modification of standard FM-UWB, multi-channel transmission.

All of the aforementioned techniques could prove to be useful in future applications. The SC-FDMA allows multiple transmitters to transmit either to a single receiver or to different receivers at the same time, without a significant performance loss. It can also be combined with the multi-channel transmission to speed up communication in both directions. Finally, a relatively simple configurable transceiver could be implemented that would allow for trading a number of channels for the data rate per channel in order to conform to the specific needs of the application. The methods described here could eventually provide the basis for implementation of more advanced techniques at higher levels of abstraction, such as, for example, channel hopping [30], and generally lead to more optimal system performance.

## Figures and Tables

**Table 1:** Performance summary of state-of-the-art Frequency Modulated Ultra-Wideband (FM-UWB) receivers.

Reference	[6]	[9,16]	[17]	[12]	[13]	[10,18]	[7,19]
Year	2009	2010	2012	2013	2014	2014	2016
Demodulator	RF-DL	Reg	RF-DL	DBPF	DBPF	Regen.	BB-DL
Frequency [GHz]	7.5	3.75	3.8	3.75	8	4	4
Power cons. [mW]	9.4	2.2	7.2	3.8	0.6/4 *	0.58	0.42
Supply [V]	1.8	1	1.6	1	1	1	1
Data rate [kb/s]	50	100	50	100	1000	100	100
Sensitivity [dBm]	−88	−84	−70	−78	−76	−80.5	−70
NB SIR [dB]	−25	−30	-	-23	-	−18	−17
SC-FDMA	Yes	No	-	No	No	No	Yes
Efficiency [nJ/b]	188	2.2	144	38	1	5.8	4.2
Tech. node [nm]	250	65	180	65	65	65	65

^*^ Power consumption is 0.6 mW with duty-cycling and 4 mW without duty-cycling.

**Table 2:** Performance summary of state-of-the-art Frequency Modulated Ultra-Wideband (FM-UWB) transmitters.

Reference	[23]	[21]	[22]	[17]	[24]	[13]	[10,18]
Year	2010	2011	2011	2012	2013	2014	2015
SC Modulation	2-FSK	2-FSK	2-FSK	2-FSK	2/4/8-FSK	2-FSK	2-FSK
Frequency [GHz]	3.8	3.8	4	3.8	3.75	8	4
RF Bandwidth [MHz]	600	700	500	560	500	500	500
Power cons. [mW]	9.6	18.2 *	0.9	8.7	1.14	0.39/3.5 **	0.63
Supply [V]	1.6	1.6	1	1.6	1	1	1
Data rate [kb/s]	10	1000	100	50	750	1000	100
Out. power [dBm]	−14.5 ***	−12.8	−10.2	−13.7	−14	−11 ***	−10.1
Energy Efficiency	960	18.2 *	9	174	1.5	0.39	3.1
Tech. node [nm]	180	180	90	180	65	65	65

^*^ Excluding the output PA; ^**^ With and without duty cycling; ^***^ Estimated from figure.
